# Subsolid pulmonary nodule morphology and associated patient characteristics in a routine clinical population

**DOI:** 10.1007/s00330-016-4429-9

**Published:** 2016-06-02

**Authors:** Onno M. Mets, Pim A. de Jong, Ernst Th. Scholten, Kaman Chung, Bram van Ginneken, Cornelia M. Schaefer-Prokop

**Affiliations:** 1 Radiology, University Medical Center Utrecht, Utrecht, The Netherlands; 2 Diagnostic Imaging Analysis Group, Radboud University Nijmegen Medical Centre, Nijmegen, The Netherlands; 3 Radiology, Meander Medical Center, Amersfoort, The Netherlands

**Keywords:** Solitary pulmonary nodule, Ground-glass, Adenocarcinoma, Computed tomography, Disease management

## Abstract

**Objectives:**

To determine the presence and morphology of subsolid pulmonary nodules (SSNs) in a non-screening setting and relate them to clinical and patient characteristics.

**Methods:**

A total of 16,890 reports of clinically obtained chest CT (06/2011 to 11/2014, single-centre) were searched describing an SSN. Subjects with a visually confirmed SSN and at least two thin-slice CTs were included. Nodule volumes were measured. Progression was defined as volume increase exceeding the software interscan variation. Nodule morphology, location, and patient characteristics were evaluated.

**Results:**

Fifteen transient and 74 persistent SSNs were included (median follow-up 19.6 [8.3–36.8] months). Subjects with an SSN were slightly older than those without (62 vs. 58 years; *p* = 0.01), but no gender predilection was found. SSNs were mostly located in the upper lobes. Women showed significantly more often persistent lesions than men (94 % vs. 69 %; *p* = 0.002). Part-solid lesions were larger (1638 vs. 383 mm^3^; *p* < 0.001) and more often progressive (68 % vs. 38 %; *p* = 0.02), compared to pure ground-glass nodules. Progressive SSNs were rare under the age of 50 years. Logistic regression analysis did not identify additional nodule parameters of future progression, apart from part-solid nature.

**Conclusions:**

This study confirms previously reported characteristics of SSNs and associated factors in a European, routine clinical population.

***Key Points*:**

• *SSNs in women are significantly more often persistent compared to men*.

• *SSN persistence is not associated with age or prior malignancy*.

• *The majority of (persistent) SSNs are located in the upper lung lobes*.

• *A part-solid nature is associated with future nodule growth*.

• *Progressive solitary SSNs are rare under the age of 50 years*.

## Introduction

Subsolid pulmonary nodules (SSN) are a special subgroup of lung nodules with specific characteristics distinct from solid nodules, especially regarding growth rate and malignancy risk [1–4]. SSNs are either transient or persistent, and represent benign causes such as infection or focal fibrosis on the one hand, or (pre)malignant lesions lesions from the adenocarcinomatous spectrum on the other [5–7]. Regardless of the final aetiology, every new and persistent SSN is an indication for surveillance. Advances in CT technology and emerging data on the meaning of this entity have increased awareness and knowledge substantially in recent years [7]. Recently, guidelines are issued for SSN management [8, 9] based on the currently available data. Briefly, this management mainly involves imaging surveillance over several years, based on nodule characteristics (i.e. pure ground-glass or part-solid nature) and growth/transformation over time. If a malignancy is suspected, additional examinations including biopsy, resection, and/or other therapy is recommended in a multidisciplinary approach, based on the individual patient characteristics and preferences [8, 9].

Although the available guidelines are meant to be applied in clinical practice, most available data on SSNs originate from lung cancer screening subjects, a different setting that is likely not representative for daily clinical practice. The currently available non-screening data comes mainly from Asian cohorts and from pathologically proven SSNs, which may introduce a selection bias [10–15]. Also, most studies focus on either pure or part-solid lesions and solely aim to identify predictors for growth, malignancy, or invasiveness [12–14, 16, 17]. Although these studies elucidate important features of SSNs, they do not provide a complete overview of the SSN entity in routine clinical practice. It was our hypothesis that clinically detected SSNs differed in their characteristics, behaviour, and/or patient characteristics from those detected in the screening setting. Therefore, we aimed to provide data on the generalizability of screening data by determining presence and morphology of SSNs in a routine non-screening setting and relate them to clinical and patient characteristics.

## Methods

The local ethical institutional review board waived the need for informed consent for this study, due to its retrospective design.

### Data collection

An automatic search was made for the description of a subsolid nodule in all reports of chest CT examinations obtained in adults in our academic hospital between June 2011 and November 2014. The dataset involved a routine clinical patient population, in whom no selection was made based on imaging indication, prior disease, or outpatient/hospitalized status. All reports that contained the terms “ground-glass”, “subsolid”, “part-solid”, or the equivalent Dutch terms were selected. “Nodule” or “lesion” was not added in the search to avoid overly narrowing the selection. One of the authors read all the selected reports to determine whether the identified terms where used to describe a nodular subsolid lesion (i.e. a possible SSN). In case of doubt, the subject was included. Subjects that originated from an ongoing lung cancer screening trial were excluded, since these did not represent routine clinical subjects. Basic clinical parameters such as gender, age, and oncologic history were obtained from the radiology information system.

Two observers (OMM and EThS; with >5 and >20 years of experience in chest CT, respectively) in consensus scored all included scans for the presence of a nodular subsolid lesion. In case of discrepancy, the case was arbitrated by a third observer (thoracic radiologist with >10 years of experience; PAJ). One observer (OMM) then reviewed all available chest CT examinations of the included cases to identify the first and last available chest CT scan with thin-slice reconstructions on which the SSN was visible. To determine longitudinal nodule dimensions, these scans were subsequently processed by the same observer using dedicated semi-automatic nodule software (CIRRUS Lung, Diagnostic Image Analysis Group, Nijmegen, The Netherlands, Fraunhofer MEVIS, Bremen, Germany). The nodule segmentation technique has previously been described in more detail, as was the interscan variation using this software [18]. A subset of 10 subjects (i.e. 20 CT scans) was analysed twice, to determine intraobserver agreement.

During nodule segmentation, nodule location and morphology (pure ground-glass or part-solid) were also annotated. In case of multiple subsolid pulmonary nodules, the largest or most dominant lesion was selected, where part-solid lesions prevailed over pure ground-glass lesions. Progression of the nodule was based on its volume increase in the two CT scans, defined as an increase ≥28.6 % using this specific software [18]. When a case showed equal or decrease in total volume, but transition into a part-solid lesion with growth of the solid component, it was also classified as a progressive SSN. Growth rate of the nodules was calculated as volume doubling time (VDT) using the formula: *VDT* = ∆*T* * *log2*/*log*[*V2*/*V1*] with T in days and V in cubic millimetres (mm^3^).

Scans were obtained on three MDCT scanners of the same vendor (16-256 slice). According to protocol the images were obtained at 100-120 kVp and 60-130 mAs, depending on body weight. Thin-slice reconstructions were made using a smooth reconstruction filter (C-filter, Philips). Analyzed reconstructions had a slice thickness of ≤1 mm. Because of differences in scan protocol regarding administration of intravenous contrast we were unable to analyse longitudinal changes in nodule mass, as the increased nodule density due to the enhancement would largely influence the results when compared to the same nodule in a non-enhanced CT.

### Data analysis

Continuous data is reported as median with interquartile range (IQR), unless stated otherwise. Comparison of continuous data was performed with the Mann Whitney-U test, while proportions were compared using the Chi-square test or Fisher exact test, as appropriate. Intraobserver agreement was calculated using the intraclass correlation coefficient (ICC). Logistic regression analysis was used to determine predictors for future progression, using progression as outcome variable and patient and lesion characteristics as potential predictors. A *p*-value <0.05 was considered statistically significant.

## Results

### Data collection

**Fig. 1:**
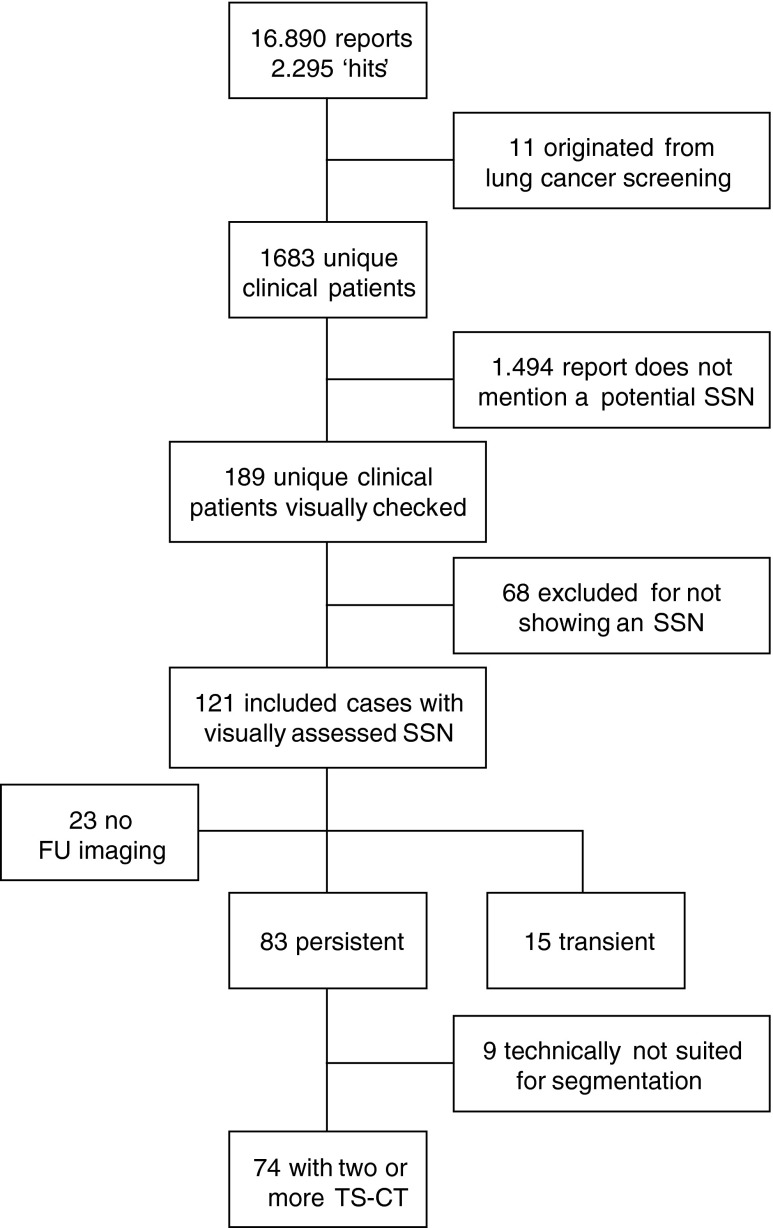
Flow chart of study population selection SSN = subsolid pulmonary nodule; FU = follow-up; TS-CT = thin-slice Computed Tomography

In the 3-year period between June 2011 and November 2014 a total of 16,890 chest CTs were obtained in 10,271 adults. In 2295 reports of 1683 unique patients at least one of the selected search terms was identified. After case inclusion based on report review, 189 remained. Visual scoring of these scans led to exclusion of 56 cases, and there was discrepancy between the two readers in 15. Refereeing by the third observer lead to exclusion of 12 of the 15 discrepant cases, leaving a total of 121 subjects with an SSN (incidence = 1.2 %). Collecting available imaging led to further exclusion of 23 cases, in whom only a single chest CT was obtained in the study period. Of the remaining 98 cases, 83 proved persistent, while 15 disappeared on follow-up. Of the 83 persistent SSNs, four subjects had only one CT scan of which the thin-slice data was stored, impeding longitudinal volume quantification. In 5/79 of the semi-automatically processed persistent SSNs, it proved technically impossible to reliably segment the SSN due to a central localisation, scan quality, or breathing artefacts. Therefore, our final study population comprised 89 subjects with 74 persistent and 15 transient SSNs. Figure 1 shows the flow-chart of the inclusion process.

### Subsolid pulmonary lesions

The total population that underwent chest CT in the study period had a median age of 58 (45 – 68) years, range 18 to 99. The subpopulation with an SSN (*N* = 121) was slightly older (61.8 [52.4 – 69.6] years, *p* = 0.01). There was no difference in gender distribution for those with and without an SSN (M/F ratio 62/59 vs. 6035/4236; *p* = 0.10). The excluded SSN subjects (*N* = 32) did not differ in age (*p* = 0.49), but were more often male (23/9 vs. 39/50, *p* < 0.01) compared to the included subjects. Table 1 summarizes the characteristics of the included study subjects.

**Table 1:** Study population characteristics

	Total (*N* = 89)	Transient (*N* = 15)	Persistent (*N* = 74)
Gender, N (%)			
Male	39 (44)	12 (80)	27 (36)
Female	50 (46)	3 (20)	47 (64)
Age, years	61.6 [52.6–68.7]	56.6 [48.8–65.1]	62.0 [53.3–68.9]
Previous malignancy			
Yes	45 (50)	5 (33)	40 (54)
No	44 (50)	10 (67)	34 (46)
Subtype			
GGN	63 (71)	11	52 (70)
PS	26 (29)	4	22 (30)
Multiplicity			
Solitary	60 (67)	12 (67)	48 (65)
Multiple	29 (33)	3 (33)	26 (35)
Location, N (%)			
RUL	32 (36)	7 (47)	25 (34)
RML	4 (4)	2 (13)	2 (3)
RLL	11 (12)	1 (7)	10 (13)
LUL	33 (37)	3 (20)	30 (41)
LLL	9 (10)	2 (13)	7 (9)
Progressive, N (%)	35	N/A	35
Diameter, mm	-	-	10.4 [7.8–14.3]
Volume, mm^3^	-	-	1530 [589–3220]

### Transient versus persistent

Comparing the transient SSNs to the persistent nodules, there was no significant difference in age (*p* = 0.22), location (*p* = 0.54), history of malignancy (*p* = 0.14) or multiplicity (*p* = 0.37). However, women showed significantly more often persistence of SSNs (47/50 vs. 27/39; *p* = 0.002).

### Persistent subsolid lesions

**Fig. 2:**
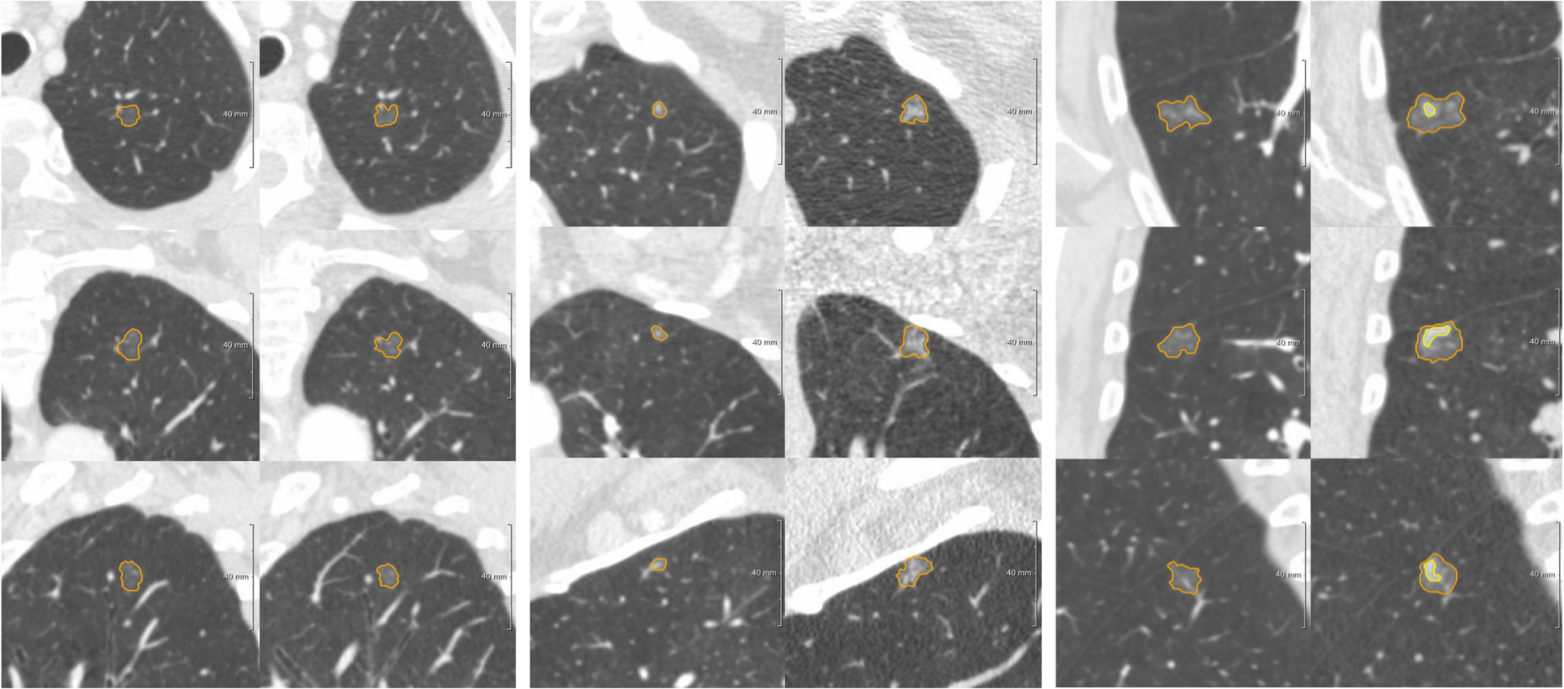
Examples of a stable GGN (left), progressive GGN (middle), and transformation of a GGN into a part-solid lesion over time (right). Panels are axial, coronal, and sagittal projections for upper, middle, and lower panels, respectively. (Left) Diameter/Volume 1 = 9.0 mm/382 mm^3^ Diameter/Volume 2 = 8.6 mm/338 mm^3^ Volume change (25.1 months) = −12 % (Middle) Diameter/Volume 1 = 5.3 mm/79 mm^3^ Diameter/Volume 2 = 11.1 mm/716 mm^3^ Volume change (36.8 months) = 803 % (Right) Diameter/Volume 1 = 13.5 mm/1281 mm^3^ Diameter/Volume 2 = 16.4 mm/2287 mm^3^; Solid component: 6.2 mm/122 mm^3^ Volume change (20.4 months) = 79 %

In the persistent SSN group, follow-up was available over a median period of 19.6 (8.3 – 36.8) months. Initially, 52 were pure ground-glass nodules (GGN), while 22 were part-solid lesions. In total 35 SSNs were classified as progressive, either due to increase in total lesion volume (*N* = 28) or due to development (*N* = 3) or growth of the solid component (*N* = 4). The other 39/74 SSNs were classified as stable over a period of 13.3 (6.0 – 27.8) months. See Fig. 2 for examples of different categories of subsolid pulmonary nodules.

Semi-automated segmentation showed near perfect intra-observer variability (ICC ≥ 0.99). Maximum diameter of the 74 nodules was at median 10.4 (7.8 – 14.3) mm, while median volume was 588.7 (244.4 – 1529.9) mm^3^. Part-solid lesions were significantly larger than pure GGN (14.6 mm/1638.3 mm^3^ vs. 9.0 mm/382.9 mm^3^; *p* < 0.001), and were more often progressive (15/22 vs. 20/52; *p* = 0.02).

Total volume change between the two CT scans was at median +18 % (IQR -8 – +38; range -49 to +1244). The 35 progressive lesions showed a median VDT of 536 days (IQR 353 – 1301, range -784 to 5691) days. Two of the progressive lesions showed a total volume decrease (VDT -784 and -660), however, with a clear growth of the solid component.

**Fig. 3:**
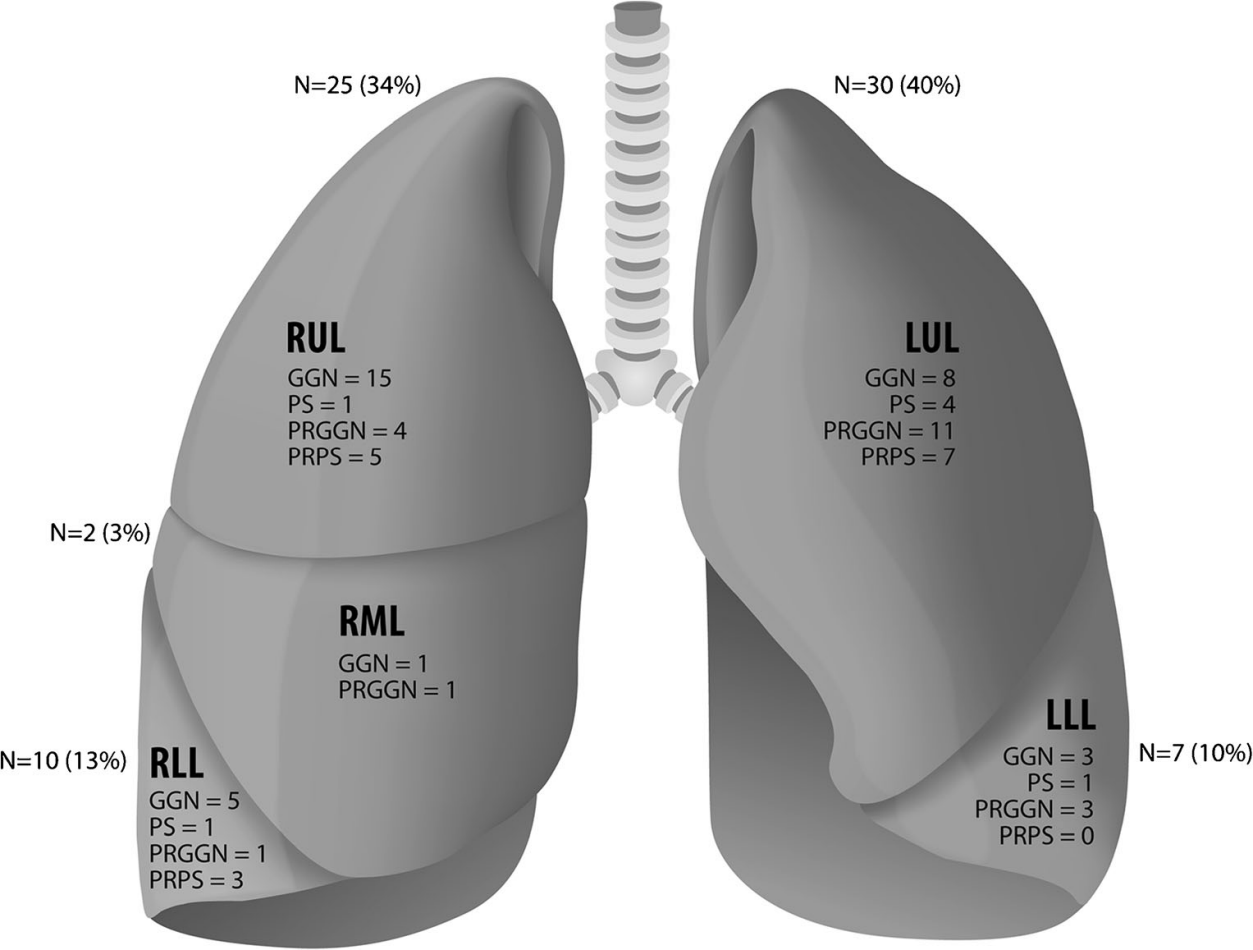
Graph schematically showing SSN distribution throughout the lungs SSN = Subsolid pulmonary nodule; RUL = Right upper lobe; RML = Right middle lobe; RLL = Right lower lobe; LUL = Left upper lobe; LLL = Left lower lobe; GGN = Ground-glass nodule; PS = part-solid nodule; PRGGN = progressive ground-glass nodule; PRPS = progressive part-solid nodule

Overall, persistent SSNs showed a clear preference for the upper lobes (55/74, 74 %), and the SSNs that were not located in the upper lobes were often found in the apical parts of the lower lobes. SSN subtype and nodule progression did not show an association with lobe distribution (*p* = 0.71 and 0.60, respectively). Figure 3 graphically shows nodule distribution throughout the lungs.

**Fig. 4:**
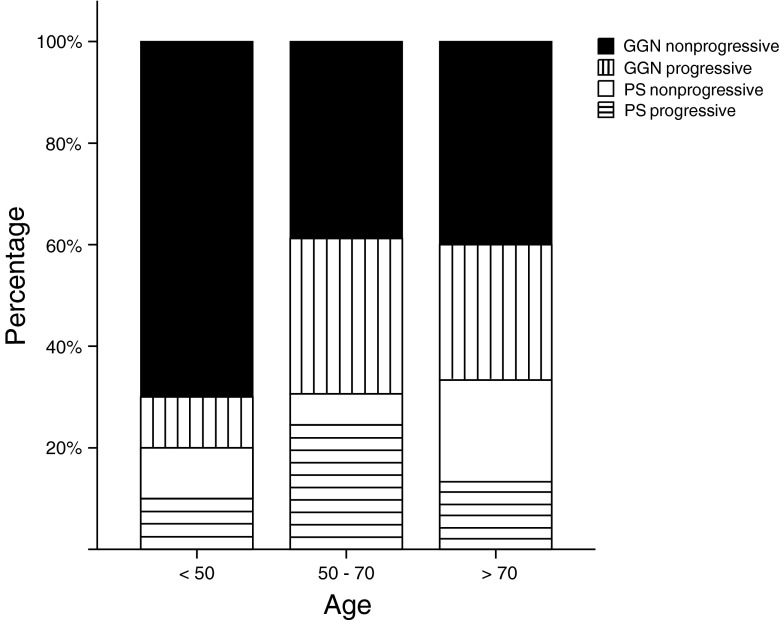
Graph showing the distribution of SSN subtypes over the different age categories. SSNs tend to be mainly of pure ground-glass morphology in the lower age categories, with increasing proportion of part-solid lesions in the higher age groups. It is further shown that the progressive lesions (*striped bars*) are mainly found in the middle (27 out of 35, 77 %) and oldest age category (6 out 35, 17 %), while they are scarce in the youngest age group. SSN = Subsolid pulmonary nodule; GGN = Ground-glass nodule; PS = Part-solid nodule

No significant difference was found for the different age groups, although the proportion of pure GGNs tends to decline with higher age while part-solid lesions are more commonly present. Further, progressive lesions were mainly found in the subjects aged 50 to 70 years (27/35, 77 %), while these subjects comprised 65 % (49/74) of the total cohort. Persistent SSNs are relatively rare under the age of 50 years (10/89); however, they are present under the age of 35 years (*N* = 2). Out of the two progressive lesions that were found in the <50 years old category, one was a 45-year-old man with multiple progressive part-solid lesions representing neuroendocrine metastases. The other was a 33–year-old man with metastasized non-seminoma who showed a nonspecific pure GGN that showed limited volume increase (31 %) in 45 months of follow-up. Solitary part-solid lesions were not found in the <50 years old subcategory. Figure 4 shows the distribution of SSN subtypes over the different age categories.

### Predictors of progression

Logistic regression analysis with nodule progression as outcome variable and nodule characteristics (subtype, lesion volume, location, and multiplicity) as potential predictors, showed that in our population only a part-solid nature was associated with future progression (*p* = 0.04). The other parameters did not reach significance.

## Discussion

We present a descriptive paper on the subsolid pulmonary nodule in a routine clinical setting obtained in a single academic institution. As most available data originates from lung cancer screening or Asian study populations, we aimed to evaluate whether the current knowledge on this relatively new entity is generalizable to other practices. Clinical data on SSN appearance and evolution over time may be of importance for an adapted and more personalized surveillance management in clinical subjects, where, for example, gender- or age-defined subgroups can be identified that require different follow-up intervals. In the current study we found that women more often show persistent lesions compared to men, and that part-solid SSNs are larger and more often progressive compared to pure GGNs. In our study a part-solid nature was the single predictor of nodule growth over time.

Because of our retrospective study design and case selection based on the radiological reports, it is challenging to comment on the true incidence of SSNs in clinical practice. In this study we found an incidence of 1.2 % (121/10.271), which is in line with available literature [9]. Nevertheless, highly variable incidence – up to 38 % – is reported in the literature, depending on the study population [7]. This high variability also applies to the proportion of transient versus persistent lesions [7]. Beyond study design and possible selection bias, the inherent characteristics of a clinical population (e.g. immune status) can influence the incidence and nodule type distribution of SSNs. This should always be kept in mind when comparing study results.

Regarding persistence, we showed that a significantly higher proportion of SSNs is persistent in women. This is in line with Lee et al., who showed that male sex was associated with the transient nature of part-solid SSNs in a screening cohort [19]. They also found that younger age and multiplicity were predictive for a transient nature; however, we found no other differences in baseline characteristics between those with a transient or persistent SSN. We believe this may well be due to either our study population size and/or the fact that we did include both types of SSNs.

The current guidelines of the Fleischner Society [8] and the British Thoracic Society [9] treat men and women alike and advise no lower age limit in their management recommendations –as is used in the management of solid pulmonary nodules– because lung adenocarcinoma also occur in younger non-smoking subjects. Comparing our findings to the current knowledge, we also did not find a difference in gender distribution among clinical subject with an SSN. Regarding age, our study shows that persistent SSNs are relatively rare in patients less than 50 years of age. When they do occur, we found mostly non-progressive pure GGNs. However, our study has limited follow-up and, therefore, some of these lesions potentially represent lesions of more importance in the long run. Solitary lesions of short-term importance (i.e. single progressive part-solid lesion, or transformation of a ground-glass nodule into a part-solid lesion) were not present in our cohort < 50 years. Progression was only seen in cases of multiple lesions caused by metastases, in patients that are already under surveillance. In the middle aged patients (i.e. 50-70 years of age), on the other hand, progressive lesions were far more common; over half of the SSNs in this subgroup showed progression during follow-up. Also, the GGN vs. part-solid ratio tends to tip more to the latter with increasing age. Taken together, these findings suggest that the worrisome SSNs are mainly found in the aging lung and that age-dependent surveillance intervals might well be appropriate. However, our results are preliminary and future studies and pooling of data could further elucidate this issue to determine reliably surveillance subgroups. A strategy that potentially can lower the number of CT scans and cumulative radiation exposure in long-term follow-up, especially in younger subjects. Another interesting finding was that nearly three quarters of the SSNs were located in the right or left upper lobe. This same pattern of lobar distribution for SSNs was present in previous study reports [16, 20], however, was not further explored. We now did not find an association between nodule distribution and persistence, SSN subtype or progression. Knowledge on upper lobe preference might support and focus observers in SSN detection, but given the absence of clear distribution patterns with regard to SSN morphology, it is unlikely to be of major influence in adaptation of surveillance management and is rightfully not part of the current guidelines.

Nevertheless, the preference for the upper lobes might suggest a relation with inhalation/smoking, as is described for lung cancer, which is clearly most prevalent in the right upper lobe [21]. Unfortunately, we cannot explain the upper lung location preference with certainty and are unable to determine a possible relationship with smoking as detailed smoking history is unknown in our clinical cohort. In general, limited data are available on this issue [8, 9].

In the search for factors associated with future progression of the nodule, we found only the part-solid nature of SSNs to be associated. This is an already well known and extensively reported parameter [11, 16, 20, 22] together with initial lesion size [11, 12, 16, 20, 22, 23], which is fundamental for the current management guidelines. We feel that the failure to reproduce other previously reported predictors of progression is likely due to our study population characteristics and relatively small study size.

The strength of our study is that to our best knowledge we are the first to present a descriptive study of SSN morphology in a non-Asian and routine clinical population, not focused on a specific SSN subtype and without selection on pathological confirmation. Further, we have used semi-automated software to determine which SSNs were progressive over time, instead of using less reliable visual scores. Our study also has some limitations. First, it is a retrospective, single-centre study, in which we may have selected cases under possible reporting bias. However, this is inherent to the current study design and difficult to avoid when larger cohorts of SSNs are required. Second, the study population is fairly small, which may either reflect the low prevalence of these type of nodules or a reporting bias. Nevertheless, we were able to include a sufficient number of both GNN and part-solid SSNs with longitudinal imaging data, not largely different from previous studies. Third, given the retrospective analysis in a routine clinical population we had no conclusive data on risk factors for lung cancer such as family history or detailed smoking history. Also, scan protocols regarding intravenous contrast administration were not uniform, which impeded evaluation of lesion mass. Additional to contrast injection, quantitative measurements of subsolid nodules can be influenced by several other factors including reconstruction protocol, dose, and tube voltage. Nevertheless, phantom experiments suggest that the influence is limited [24]. In our study we did not change tube voltage. There are currently no data on the effect of contrast injection for volume measurements of subsolid nodules, but for solid nodules the volume difference is about 15 % [25]. Last, we do not have pathological confirmation of the pulmonary nodules, which precludes conclusions on the prevalence of (pre)malignancies in our subgroups. Nevertheless, we did not introduce a selection bias by selecting only proven malignancies and reflect daily practice in which unconfirmed lesions are encountered and surveyed.

In conclusion, our descriptive study provides complementary data on SSN morphology in a European, routine clinical population. We confirm several previously reported characteristics obtained in other study populations and provide additional data on subject age and lung distribution of SSNs. Although our results are preliminary, these data might contribute to a more individualized approach of SSN management in future routine clinical practice.

## Abbreviations

SSN: Subsolid nodule
GGN: Ground-glass nodule
PS: Part-solid nodule
CT: Computed Tomography
FU: Follow-up
VDT: Volume doubling time
